# Sexual Consent in Committed Relationships: A Dyadic Study

**DOI:** 10.1080/0092623X.2021.1937417

**Published:** 2021-07-19

**Authors:** Malachi Willis, Kelli N. Murray, Kristen N. Jozkowski

**Affiliations:** a Institute of Health and Wellbeing, MRC/CSO Social and Public Health Sciences Unit, University of Glasgow, Glasgow, UK; b Department of Health, Human Performance, and Recreation, University of Arkansas, Fayetteville, AR, USA; c Department of Applied Health Science and the Kinsey Institute for Research in Sex, Gender and Reproduction, Indiana University, Bloomington, IN, USA

## Abstract

Sexual consent is a multidimensional construct that requires the participation of all involved in a sexual encounter; however, previous research has almost exclusively relied on one person’s perspective. To address this, we collected open- and closed-ended data on sexual consent from 37 dyads in committed sexual relationships (*N* = 74). We found that relationship length was associated with sexual consent and couples who accurately perceived each other’s consent communication cues reported elevated levels of internal consent feelings. Communicating willingness to engage in sexual activity remains important even within committed relationships. Preliminary findings suggest that further investigations of dyadic nuances of sexual consent are warranted.

## Introduction

Sexual consent refers to people’s willingness to engage in partnered sexual activity. Typically, one person initiates or requests a sexual behavior, and another person responds based on their willingness to take part in that sexual behavior (Muehlenhard et al., 2016). This process of sexual consent can be iterative, building toward and continuing throughout a consensual sexual encounter (Beres, 2014; Humphreys, 2004; Willis & Jozkowski, 2021). Given these interactive components, consensual sex cannot exist based on the sole willingness of one person. However, to our knowledge, empirical investigations of sexual consent have almost exclusively assessed only one person’s perspective. In the present study, we sought to fill this gap by examining sexual consent in a sample of dyads who were in committed sexual relationships—an important context to consider given normative beliefs that assuming the sexual consent of committed partners is okay (O’Byrne et al., 2008; Righi et al., 2019; Willis & Jozkowski, 2019).

## Defining sexual consent

Broadly defined, sexual consent comprises three distinct aspects: feelings, communication, and perceptions (Muehlenhard et al., 2016). First, sexual consent can be conceptualized as an internal state of willingness to engage in sexual activity with another person (Jozkowski, Sanders, et al., 2014). Second, people can communicate that willingness to others using words and behaviors; either of which may be explicit or implicit (Hickman & Muehlenhard, 1999). Third, people need to perceive the communicative cues of others—or cues based on context—to determine whether that other person is willing. We assessed each of these aspects of sexual consent to provide a comprehensive account of sexual consent within sexual and romantic dyads.

Whether somebody is willing to engage in a particular behavior with a particular person within a particular context depends on a multidimensional process of internal feelings.

To assess the breadth of feelings associated with sexual consent, one research team asked participants to write about the feelings that they associate with being willing to engage in sexual activity (Jozkowski, Sanders, et al., 2014). These researchers ultimately developed a scale that comprised five feelings related to internal consent: physical response, safety/comfort, arousal, agreement/want, and readiness. This scale was developed to differentiate internal consent feelings specifically within the context of consensual sexual encounters.

Because people are not intuitively privy to the feelings of others, sexual partners typically find ways to let each other know that they feel ready, safe, aroused, desirous, and physically responsive. Best practice entails partners actively communicating their consent to sexual activity because active cues are positively associated with people’s internal consent feelings (Jozkowski, Sanders, et al., 2014; Walsh et al., 2019). Active consent communication involves partners doing or saying something to indicate their willingness to engage in sexual activity; people’s actions and words can be straightforward or subtle. This two-by-two system of categorization (i.e., verbal vs. nonverbal; explicit vs. implicit) was proposed by Hickman and Muehlenhard (1999), who also discussed a fifth “no response” cue that captures when people passively communicate their consent by letting sexual activity happen without saying anything or without resisting. Although no response cues are commonly perceived to be indicators of consent (Jozkowski, Sanders, et al., 2014; Willis, Hunt et al., 2019), such passive communication does not reliably reflect people’s willingness to engage in sexual activity and, thus, should not be used to infer sexual consent (Willis, Blunt-Vinti, et al., 2019).

Research indicates that nonverbal consent cues are used more frequently than verbal cues (Hickman & Muehlenhard, 1999; Orchowski et al., 2020; Righi et al., 2019). Despite the potential subtlety of nonverbal cues, qualitative evidence suggests that people are deft communicators when it comes to sex, effectively discerning their partners’ hints and behaviors as willingness or refusal (Beres, 2014; O’Byrne et al., 2008). As a result, explicit verbal consent communication is considered by many to be inconsistent with the cultural norms for sexual consent between partners (Burkett & Hamilton, 2012; Curtis & Burnett, 2017); however, people still acknowledge that verbal affirmation of consent tends to diminish confusion regarding their or their partner’s willingness to engage in sexual activity (Righi et al., 2019).

Communicating willingness is not unidirectional. Perceptions necessarily play an important role in the process of communicating sexual consent between two or more people. Much of the previous research on sexual consent perceptions has asked people to interpret whether fictional characters are willing to engage in sexual activity (e.g., Humphreys, 2007; Willis & Jozkowski 2021). However, work on people’s perceptions of their actual partners’ sexual consent is limited. One recent study asked women to report the communication cues they perceived their male partner had used during their most recent sexual activity (Willis, Blunt-Vinti, et al., 2019), finding that about half of the participants perceived their partners had used explicit verbal or implicit verbal cues. Another study asked participants to describe the “words or behaviors [they] would look for from [their] partner to indicate that he/she was willing … to have sex with [them]” (Jozkowski, Peterson, et al., 2014, p. 908). Similar to research on people’s own consent cues, participants in that study indicated that they perceived their partner’s willingness based on communication that was verbal, nonverbal, or lacked a refusal.

In sum, sexual consent reflects an interpersonal process that is iterative and cyclical (Humphreys, 2004; Willis & Jozkowski, 2021; Muehlenhard et al., 2016). One person may begin by feeling willing to engage in a sexual behavior with another person, so they try to communicate their willingness to the other person in some manner and consequently look for indications that the other person is also willing. Meanwhile, the other person is simultaneously navigating each of these three aspects of sexual consent themselves. This process continues throughout the duration of a consensual sexual encounter; however, previous research has been limited by systematically focusing on one side of this interaction. To our knowledge, no studies have collected data from dyads to assess whether people are able to accurately perceive the consent communication that their partners self-report using.

## Sexual consent and gender

According to traditional sexual scripts, people who identify as women are more likely to be the gatekeeper in a given encounter and thus accept or rebuff a male initiator’s attempt for sex (Curtis & Burnett, 2017; Jozkowski et al., 2017; Wiederman, 2005). Based on these stereotypically gendered roles, both women and men tend to describe sexual consent as something men get from women (Hirsch et al., 2019; Pugh & Becker, 2018; Righi et al., 2019). Perhaps not surprisingly, previous research has shown that gender may be associated with people’s sexual consent feelings, communication, and perceptions.

First, existing literature is mixed but generally indicates that gender differences regarding internal consent depend on the feeling in question. For example, Jozkowski, Sanders, et al. (2014) found that women reported lower levels of arousal and higher levels of safety and comfort than men; however, a different study found that women scored higher on physical response (Walsh et al., 2019). Further, traditional sexual scripts suggest that men are expected to always want sex (Murray, 2018), which may affect how they internalize and report their feelings of willingness to engage in sexual activity.

Second, women tend to communicate their willingness to engage in sexual activity indirectly—likely due to being socially reinforced as gatekeepers and experiencing inhibited sexual agency (i.e., ability to act on one’s own behalf sexually, express needs and desires, and advocate for oneself; Tolman et al., 2015). Evidencing gender differences in sexual consent communication, studies have found that men were more likely than women to use explicit verbal cues relative to implicit nonverbal cues (Willis, Hunt, et al., 2019), whereas women were more likely to let sexual behaviors happen to them without resisting (Jozkowski et al., 2017; Walsh et al., 2019).

Third, gender is associated with the cues people use to interpret another person’s willingness. In Jozkowski, Peterson, et al.’s (2014) study, women relied significantly more on verbal cues to perceive consent, while men tended to look for nonverbal cues from their partners. In an experimental vignette study, men were more likely than women to perceive that a fictional woman was willing to engage in sexual behavior when she initiated transitioning from a public to a private setting—compared with conditions in which a fictional man initiated the transition (Jozkowski & Willis, 2020). Based on these previous findings, gender remains an important construct to consider in sexual consent research.

## Sexual consent within committed relationships

Each aspect of sexual consent—feelings, communication, and perceptions—can also depend on the context in which sexual encounters occur (Willis & Jozkowski, 2019). One context that has consistently been considered in the academic literature on sexual consent is relationship status. Evidencing that people’s beliefs about sexual consent vary based on relational context, participants in Humphreys’ (2007) vignette study agreed more with the phrase “sexual consent is okay to assume in this context” when the characters were in a committed relationship versus a first-time or casual encounter—even though the consent communication cues presented in each condition were the same (p. 310). Indeed, simply being in a committed relationship with somebody can be perceived as a contextual cue for consent (O’Byrne et al., 2008; Righi et al., 2019). Thus, people believe that being in a committed sexual relationship with a partner can decrease the need to communicate consent explicitly.

Even though people seem to believe sexual consent can be assumed within the context of a committed sexual relationship (Humphreys, 2007; O’Byrne et al., 2008), research indicates that those in committed relationships are actually more likely to use verbal consent cues than those in casual relationships (Marcantonio et al., 2018; Willis, Hunt, et al., 2019). One potential explanation for this finding is that people in committed relationships—compared with those in casual relationships—may be more comfortable explicitly and verbally communicating their consent because they feel confident interpreting a romantic partner’s signals (Righi et al., 2019) or do not fear rejection from them (Foubert et al., 2006).

Regarding internal consent, people in committed relationships consistently report elevated feelings of consent compared with those in casual relationships. Walsh et al. (2019) found that increasing level of intimacy with one’s partner was associated with higher levels of internal consent feelings; dating partners and significant others had the highest scores, followed by friends, acquaintances, and people who had just met. This trend was significant for each of the subscales: physical response, safety/comfort, arousal, agreement/want, and readiness. In a study that compared first-time, casual, and serious partners, safety/comfort, agreement/want, and readiness were associated relationship status; again, more intimate relationships had higher levels of these internal consent feelings (Marcantonio et al., 2018). Finally, Jozkowski, Sanders, et al. (2014) found that relationship status (i.e., single versus in a committed relationship) was associated with feelings of safety/comfort.

Most of the extant research on the association between relationship status and sexual consent has focused on comparisons between committed relationships and casual relationships; much less attention has been given to the variability of sexual consent within the context of committed relationships. Looking at this potential variability within committed relationships, a recent study examined the effect of sexual precedent (i.e., a person’s sexual history with somebody else) on people’s sexual consent communication (Willis & Jozkowski, 2019). Using a sample in which 86.9% of participants were in an exclusive, monogamous relationship, these researchers found that participants who had increasingly established sexual histories with their partners less frequently relied on active communication cues—whether verbal or nonverbal—to determine sexual consent and more frequently assumed consent based on contextual cues, such as perceiving their relationship status or feelings of love for their partner as indicators of consent. Unfortunately, Willis and Jozkowski (2019) did not measure other nuances such as relationship length. As such, additional research on how aspects of sexual consent might vary across committed relationships is warranted.

## Present study

We aimed to extend the current literature on sexual consent in two ways. First, previous research on sexual consent has relied almost exclusively on the perspective of one person; thus, how accurately^1^ people perceive the consent communication of their partners remains unknown. We overcame this limitation by collecting data from dyads in committed relationships. Second, extant research on the variability of sexual consent across committed relationships is lacking. In our sample of dyads, we addressed this gap by assessing how the length of a committed relationship might be associated with sexual consent. Given the present study’s novelty, we pursued the following research questions in an exploratory manner.

**RQ1**: How do people in committed sexual relationships describe the way they typically communicate their sexual consent?
**RQ1a:** Are gender and relationship length associated with how people in committed sexual relationships typically communicate their sexual consent?
**RQ2**: How do people in committed sexual relationships feel, communicate, and perceive sexual consent at the event-level?
**RQ2a:** Are gender and relationship length associated with event-level sexual consent feelings, communication, or perceptions?
**RQ3**: Within a committed sexual dyad, how accurate are a person’s self-reported event-level consent communication cues with their partner’s perceptions of their cues at the event-level?
**RQ3a:** Is event-level accuracy of consent perceptions associated with event-level consent feelings?

## Method

### Participants

We recruited 37 dyads (*N* = 74) to participate in this study. Data from all 37 dyads were included in our assessment of participants’ typical consent communication. However, eight dyads were excluded from the event-level analytic sample for not referencing the same partnered sexual event when responding to items in the survey; another one dyad was excluded because one participant in the dyad did not respond to key constructs. Thus, the event-level analytic sample included 28 dyads (*n* = 56). Table 1 presents the sociodemographic characteristics for the both full sample and event-level subsample.

**Table 1.** Sociodemographic characteristics

Individual variables	Full sample (*N* = 74)	Event-level subsample (*n* = 56)
**Age in years**		
*M* (*SD*)	22.4 (3.9)	22.6 (4.2)
**Gender (%)**		
Woman	40 (54.1)	29 (51.8)
Man	33 (44.6)	27 (48.2)
Other	1 (1.4)	0 (0.0)
**Race/Ethnicity (%)**		
White	56 (75.6)	41 (73.2)
Asian	3 (4.1)	3 (5.4)
Black	3 (4.1)	2 (3.6)
Multiracial/Other	12 (16.2)	10 (17.9)
**Student status (%)**		
Undergraduate	50 (67.6)	37 (66.1)
Graduate	11 (14.9)	8 (14.3)
Non-degree seeking	1 (1.4)	1 (1.8)
Not a student	12 (16.2)	10 (17.9)
Dyadic Variables	(*N* = 37)	(*n* = 28)
**Relationship length in months**		
*M* (*SD*)	38.3 (26.8)	37.1 (28.7)
**Gender of partners (%)**		
Woman-Man	33 (89.1)	27 (96.4)
Woman-Woman	3 (8.1)	1 (3.6)
Woman-Other	1 (2.7)	0 (0.0)
**Relationship type (%)**		
Exclusive/monogamous	35.5 (95.9)	27.5 (98.2)
Non-exclusive/non-monogamous	1 (2.7)	0 (0.0)
Mainly casual	.5 (1.4)	.5 (1.8)

### Procedure

Using physical flyers and a campus-wide e-newsletter at a southern university in the United States, we recruited participants to “fill out a questionnaire about their sexual experiences.” To be eligible for this study, participants had to be at least 18 years old and in a committed relationship in which they had ever previously engaged in oral sex or vaginal intercourse.^2^ Further, both the interested person and their partner had to be willing and able to participate in the study at a laboratory setting on campus. Interested people emailed the lab, and those who met all participation criteria were invited to schedule a time at which they and their partner could simultaneously participate in the study.

The second author met with each dyad in a common area at the scheduled time and guided them to a lab setting where the participants separately completed the in-person survey, which was designed to take 30 minutes to complete. Each computer was assigned a systematic identification number before entry to ensure that responses for participants in each dyad could be paired while remaining anonymous. In the lab setting, the second author distributed and explained the consent forms to both participants, who individually indicated their willingness to participate before beginning the study. Participants were seated at a computer with their backs toward each other. They were instructed to silence their phones and place them on the desks. The second author sat in the lab space while the survey was completed online via Qualtrics Survey software to eliminate the possibility of participants communicating their answers to each other; blinders were added to computers to keep the screens out of the researcher’s view.

Upon completing the study, participants each received $10USD for their participation. Finally, participants were debriefed regarding the purpose of the study and asked to not disclose the questions on the survey to others. The procedure for this study was approved by the university’s institutional review board.

### Measures

#### Typical sexual consent communication

To assess how people in committed relationships typically communicate their sexual consent, we asked participants, “In your own words, how do you and your current partner typically indicate that you are willing to engage in sexual activity?” Participants then provided their open-ended responses. Across participants, responses were about 31 words long on average with a median of 24 words and range of 3 to 179 words.

#### Event-level sexual activity

To increase the likelihood that both partners in a committed sexual relationship were referring to the same sexual experience when completing the survey, we asked participants: “Please think of the last time you and your partner engaged in vaginal intercourse. When did this happen?” All dyads in the event-level analytic sample had previously engaged in vaginal intercourse; otherwise, they would have been asked about the most recent time they engaged in oral sex. They then recorded their response on a virtual calendar. Only dyads in which both partners indicated they had last engaged in vaginal intercourse on the same day were included in the analytic sample (adjacent dates were considered to be the same day).

#### Event-level sexual consent feelings

To assess internal feelings of sexual consent, we administered the Internal Consent Scale (ICS; Jozkowski, Sanders, et al., 2014). Participants indicated the extent that they had experienced feelings related to sexual consent during their most recent vaginal intercourse event with their committed partner. Response options were on a four-point scale (“Strongly disagree” to “Strongly agree”). Higher values indicate stronger feelings of internal consent.

This scale has twenty-five items and five factors: physical response (e.g., “I felt heated”), safety/comfort (e.g., “I felt secure”), arousal (e.g., “I felt turned on”), agreement/want (e.g., “The sexual act itself felt desired”), and readiness (e.g., “I felt sure”). The ICS demonstrated strong internal reliability across these factors in the present study (αs ≥ .86) as well as its validation study (αs ≥ .90; Jozkowski, Sanders, et al., 2014), and its robust measurement properties have been replicated in multiple samples (Walsh et al., 2019; Willis, Blunt-Vinti, et al., 2019). Four participants were missing data for one item; these cells were replaced with the mean of the participants’ responses to the other items in the same subscale.

#### Event-level sexual consent communication

To assess external consent communication, we asked participants to indicate how they communicated sexual consent during their most recent vaginal intercourse event with their committed partner. We administered five items used by Willis, Blunt-Vinti, et al. (2019) to reflect the five consent techniques identified in previous research (Hickman & Muehlenhard, 1999). To report their own behaviors, participants could select “I used direct verbal cues such as saying I want to have sex,” “I used indirect verbal cues (like hints) such as asking my partner to get a condom,” “I used direct non-verbal cues such as just starting to do the behavior (e.g., moving my partner’s hands toward my genitals; starting to have sex),” “I used indirect non-verbal cues such as making eye contact or touching my partner’s arm, back, or legs,” or “I let the behavior happen without resisting or stopping it” (i.e., no response). As operationally defined, these types of consent cues were not mutually independent; participants could select multiple cues if they applied. Responses were coded dichotomously: 1 = endorsed the cue; 0 = did not endorse the cue.

#### Event-level contextual consent cues

We asked participants how else they knew that their partner was willing during their most recent vaginal intercourse event (i.e., other than communication cues). We provided a list of 12 relationship-based contextual consent cues based on previous research (e.g., Willis & Jozkowski, 2019). Examples included “We love each other” and “We have had sex before.” Participants were instructed to select all contexts that they used as indicators of their partner’s willingness during their most recent event.

#### Event-level sexual consent perceptions

To assess participants’ interpretation of their committed partner’s sexual consent cues, we also asked them to indicate how their partner had communicated consent during their most recent vaginal intercourse event. They could select from the same five types of consent communication cues. Similarly, as operationally defined, these types of consent cues were not mutually independent; participants could select as many cues as applied. Responses were coded dichotomously: 1 = endorsed the cue; 0 = did not endorse the cue.

Further, we created scores to indicate whether participants within a dyad accurately perceived their partner’s self-reported use of sexual communication cues and whether partners accurately perceived a participant’s self-reported cues: 1 = accurate perception; 0 = inaccurate perception. Scores were created for each type of consent cue measured. For example, if a participant perceived their partner had used explicit verbal cues and that partner self-reported they had used explicit verbal cues, then we scored this participant as having accurately perceived their partner’s use of explicit verbal cues.

#### Event-level orgasm

Because researchers have suggested that sexual consent may be associated with constructs related to sexual pleasure (Marcantonio et al., 2020), we asked participants how many orgasms they experienced during their most recent vaginal intercourse event. Responses were categorized (i.e., 0, 1, 2+).

### Analysis

#### Open-ended analysis

We coded the open-ended data regarding typical sexual consent communication by drawing on themes from previous research (e.g., Hickman & Muehlenhard, 1999; Jozkowski et al., 2016). To create the codebook for this question, the first and third authors independently read separate subsamples of the responses (∼25% in total) to identify salient statements that were relevant to the theme of sexual consent communication (Braun & Clarke, 2006). These authors then met to discuss the relevant responses, create codes, and draft operational definitions. Per Braun and Clarke’s recommendations, they then independently coded a subsample of the responses (∼15%) to test and subsequently refine our definitions.

To ensure reliability of coding, Neuendorf (2011) recommended 10–20% of the full sample of responses have multiple coders, aiming for at least 70% agreement. We exceeded this recommendation; all responses in the present study were coded by two coders, resulting in 95.5% agreement. Another test of interrater reliability (i.e., Cohen’s kappa) showed that there was strong agreement between coders for each code that was not likely due to guessing, indicating a reliable coding system. Cohen’s kappa for each code is provided in Table 2.

To test the associations of the codes for typical sexual consent communication with gender and relationship length, we conducted chi-squared tests of independence. We reported Cramér’s V (φ_C_) as a measure of effect size for each of the chi-squared tests. A φ_C_-value of .10 indicates a small effect size, .30 medium, and .50 large (Cohen, 1988).

#### Closed-ended analysis

We calculated descriptive statistics and tested bivariate correlations. All tests of significance had an α-level of .05. Data preparation and analyses were conducted in SPSS 26.

## Results

### RQ1: typical sexual consent

Based on our thematic analysis, we identified 10 themes across participants’ open-ended responses regarding how they and their partner typically communicate their willingness to engage in sexual activity with each other. Operational definitions for each theme are provided in Table 2.

**Table 2.** Operational definitions and indices of inter-rater reliability.

Variable	Operational definition	Percent agreement	Cohen’s Kappa
Verbal cues	Participants indicated that they or their partners use words (i.e., verbal cues) to communicate their willingness. Verbs that were to be considered verbal in nature included (but are not limited to) tell, ask, and say. Verbal cues could have also been coded as explicit or implicit but dido not have to be.	98.6	.952
Nonverbal cues	Participants indicated that they or their partners use behaviors or actions (i.e., nonverbal cues) to communicate their willingness. Nonverbal cues could have also been coded as explicit or implicit but did not have to be.	97.3	.924
Explicit cues	Participants indicated that they or their partners use signals that are most likely understood at face-value to communicate their willingness. These cues might be described as direct, clear, etc. Coders could have also deemed specific behaviors to be explicit.	95.9	.899
Implicit cues	Participants indicated that they or their partners use signals that suggest they are willing but are not likely understood at face-value. These cues might be described as indirect, subtle, etc. Coders could have also deemed specific behaviors to be implicit.	93.2	.835
Escalation	Participants indicated that they or their partners engage in behaviors that build toward a sexual act. These behaviors could have been sexual or non-sexual. These responses would have suggested that there is a process that precedes consensual sexual behavior.	100.0	1.000
Just Happens	Participants indicated that sexual activity “just” happens. These responses would have suggested that behaviors started without any indication of preceding communication.	95.9	.648
Refusals	Participants indicated that they or their partners would refuse a sexual advance if they were not willing, were not interested, or did not want to. As such, the implied cue is “not saying no” or “not refusing.”	93.2	.631
Self-reported cues	Participants referred to the cues they use to communicate their own willingness to their partners. These responses would have typically relied on the singular first-person pronouns like “I.”	91.9	.723
Perceived cues	Participants referred to the cues they perceive their partners use to communicate their (the partner’s) willingness. These responses would have typically relied on third-person pronouns like “she,” “he,” or “they.”	93.2	.743
Couple-centered cues	Participants referred to how they and their partner communicate their willingness together. These responses would have typically relied on the plural first-person pronouns like “we.”	95.9	.902

The first set of codes represents certain types of consent cues people described using. Specifically, participants indicated that they and their partner typically consent to sexual activity using verbal cues (82.4%; *n* = 61), nonverbal cues (75.7%; *n* = 56), explicit cues (73.0%; *n* = 61), and implicit cues (31.1%; *n* = 23). Reflecting participants’ use of words to communicate their consent, responses coded as verbal cues included “We ask if the other one wants to have sex” and “One of us might say ‘you look hot’ or something to that effect.” Regarding the use of behaviors or actions as consent indicators, nonverbal cues were represented by responses like “We usually let each other know nonverbally that one of us wants to have sex” and “We may start by rubbing the other person first.” Responses also received codes if they were explicit (i.e., direct and clear), such as “Using direct expressions we will ask if the other person wants to or not” and “Sometimes we do explicitly ask verbally.” Conversely, responses coded as implicit (i.e., indirect and subtle) included statements like “She usually begins to gently scratch my back” and “If he’s interested, he will usually stay in bed in the morning.”

In addition to the specific types of consent cues listed above, some participants described the process—or lack thereof—regarding how they and their partner typically consent to sexual activity. Relevant aspects of how people experience sexual consent included the escalation of behaviors (17.6%; *n* = 13), the sexual behavior just happening (8.1%; *n* = 6), and the presence or absence of refusals (12.2%; *n* = 9). Responses coded for behaviors building toward a sexual act included “Sometimes kissing just leads on to another thing” and “We touch each other excessively, and that leads to sex.” Regarding sexual behavior occurring apparently spontaneously, participants made statements like “It usually just happens” and “Sometimes we just end up having sex.” The presence or absence of refusals captured instances in which participants report they infer consent because neither partner has communicated that they were unwilling: “If the other is not in the mood, they will say so” or “We tell each other when we are too tired to.”

Finally, we coded the perspective participants used when describing how sexual consent is typically communicated in their relationship. Some participants (21.6%; *n* = 16) referred to how they communicate their own willingness with phrases like “I’ll usually ask if she would potentially be in the mood” and “I perform oral sex on her.” Other participants (17.6%; *n* = 13) indicated consent cues they perceive their partner uses by making statements such as “He will start gently touching to indicate that he is thinking about sex” and “My partner will typically give me cues by cleaning up her room, lighting candles, and dressing up.” But most participants (71.6%; *n* = 53) described how they and their partner communicate their willingness together; for example, “We just start touching each other” and “As a couple we are more upfront than most people.”

As seen in Table 3, only one of these themes varied significantly by gender, χ^2^(1) = 5.01, *p* = .025, φ_C_ = .26. Specifically, women (20.0%) more frequently than men (2.9%) endorsed that they or their partners would refuse a sexual advance if they were not willing, were not interested, or did not want to.

**Table 3.** Sexual consent communication codes by gender.

Consent Code	Women (*n* = 40)	Men (*n* = 34)	χ^2^	*p*	φ_C_
Verbal cues	43 (85.0%)	27 (79.4%)	0.40	.529	.07
Nonverbal cues	32 (80.0)	24 (70.6)	0.88	.347	.11
Explicit cues	27 (67.5)	27 (79.4)	1.32	.250	.13
Implicit cues	14 (35.0)	9 (26.5)	0.62	.429	.09
Escalation	7 (17.5)	6 (17.6)	0.00	.987	.00
Just Happens	2 (5.0)	4 (11.8)	1.13	.288	.12
Refusals	8 (20.0)	1 (2.9)	5.01*	.025	.26
Self-reported cues	10 (25.0)	6 (17.6)	0.59	.444	.09
Perceived cues	8 (20.0)	5 (14.7)	0.36	.551	.07
Couple-centered cues	29 (72.5)	24 (70.6)	0.03	.856	.02

*Note*. *p < .05.

Two of these themes varied by relationship length (Table 4). Only 50.0% of participants who had been in their relationship for at least 5 years described that they used nonverbal cues compared with those who had been in their relationship for less than1 year (87.5%), 1–3 years (75.0%), or 3–5 years (86.4%), χ^2^(1) = 8.32, *p* = .040, φ_C_ = .34. Further, the youngest relationships were associated with indicating that sexual activity just happens: less than 1 year (25.0%), 1–3 years (5.0%), 3–5 years (4.5%), and greater than 5 years (0.0%), χ^2^(1) = 8.17, *p* = .043, φ_C_ = .33.

**Table 4.** Sexual consent communication codes by relationship length.

Consent Code	<1 year (*n* = 16)	1–3 years (*n* = 20)	3–5 years (*n* = 22)	>5 years (*n* = 16)	χ^2^	*p*	φ_C_
Verbal cues	13 (81.3%)	17 (85.0%)	16 (72.7%)	15 (93.8%)	2.95	.399	.20
Nonverbal cues	14 (87.5)	15 (75.0)	19 (86.4)	8 (50.0)	8.32*	.040	.34
Explicit cues	11 (68.8)	15 (75.0)	15 (68.2)	13 (81.3)	1.00	.802	.12
Implicit cues	3 (18.8)	8 (40.0)	6 (27.3)	6 (37.5)	2.34	.506	.18
Escalation	4 (25.0)	4 (20.0)	3 (13.6)	2 (12.5)	1.21	.750	.13
Just Happens	4 (25.0)	1 (5.0)	1 (4.5)	0 (0.0)	8.17*	.043	.33
Refusals	1 (6.3)	4 (20.0)	3 (13.6)	1 (6.3)	2.24	.524	.17
Self-reported cues	3 (18.8)	1 (5.0)	7 (31.8)	5 (31.3)	5.56	.135	.27
Perceived cues	4 (25.0)	1 (5.0)	4 (18.2)	4 (25.0)	3.41	.333	.22
Couple-centered cues	11 (68.8)	17 (85.0)	15 (68.2)	10 (62.5)	2.61	.456	.19

*Note*.* *p* < .05.

### RQ2: event-level sexual consent

Descriptive statistics for the event-level sexual consent variables measured in this study are presented in Table 5. The average score for each type of internal consent feeling was between 3 (Agree) and 4 (Strongly Agree), indicating that most participants experienced feelings positively related to sexual consent during their most recent vaginal intercourse event with their committed partner. The most frequently endorsed sexual consent communication cue for this event was explicit verbal; approximately two-thirds of participants indicated that they had used this type of cue and that their partner had as well. Passive communication cues (i.e., no response) were the least endorsed self-reported cues for participants and perceived cues for their partners.

**Table 5.** Descriptive statistics for event-level sexual consent variables.

Variable	*n* = 56
Internal consent	*M* (*SD*)
Physical response	3.21 (.59)
Safety/comfort	3.63 (.58)
Arousal	3.65 (.61)
Agreement/want	3.67 (.51)
Readiness	3.88 (.44)
External consent (actor)	*n* (%)
Explicit verbal	38 (67.9)
Explicit nonverbal	30 (53.6)
Implicit verbal	34 (60.7)
Implicit nonverbal	32 (57.1)
No response	11 (19.6)
External consent (partner)	*n* (%)
Explicit verbal	37 (66.1)
Explicit nonverbal	31 (55.4)
Implicit verbal	31 (55.4)
Implicit nonverbal	29 (51.8)
No response	15 (26.8)
External consent (accuracy)	*n* (%)
Explicit verbal	41 (73.2)
Explicit nonverbal	37 (66.1)
Implicit verbal	31 (55.4)
Implicit nonverbal	33 (58.9)
No response	38 (67.9)

Bivariate correlations between each of the sexual consent variables are presented in Table 6. Each type of internal consent feeling was significantly associated with the other types of internal consent feelings (*r*s > .52, *p*s < .001). Further, many types of consent communication cues—self-reported or perceived—were associated with other types of cues. For example, perceiving a partner had used explicit verbal cues was negatively associated with perceiving they had used implicit nonverbal (*r* = −.39, *p* = .003) or explicit nonverbal (*r* = −.26, *p* = .049) cues. There were no significant associations between internal consent feelings and external consent communication—self-reported or perceived.

**Table 6.** Bivariate correlations between event-level internal and external sexual consent.

		1.	2.	3.	4.	5.	6.	7.	8.	9.	10.
Internal sexual consent	1. Physical response	**−.13**	**−**.05	**−**.07	**−**.12	**−**.17	**−**.22	.05	**−**.12	.09	**−**.06
	2. Safety/Comfort	.54***	**.05**	.05	.06	.02	.06	.37**	**−**.13	.14	.20
	3. Arousal	.68***	.71***	**.16**	.02	.01	**−**.08	.22	**−**.08	.14	.03
	4. Agreement/want	.54***	.68***	.66***	**−.07**	.05	**−**.03	.13	**−**.19	.10	.14
	5. Readiness	.52***	.88***	.80***	.76***	**−.04**	.14	.31*	**−**.15	.02	.08
External sexual consent	6. Explicit verbal	**−**.03	.23	.15	.16	.19	**.02**	.13	.23	.02	.05
	7. Implicit verbal	**−**.02	.06	.18	**−**.04	.03	**−**.03	**−.01**	**−**.02	**−**.01	.01
	8. Explicit nonverbal	.11	.04	.10	**−**.06	.12	**−**.24	.06	**.10**	**−**.03	.03
	9. Implicit nonverbal	.05	**−**.14	.02	**−**.03	**−**.07	**−**.13	.28*	.12	**.13**	**−**.12
	10. No response	.02	**−**.00	.08	.14	.01	.05	.01	.03	.25	**−.02**

Note. Correlations presented below the diagonal represent actor associations (i.e., association between a participant’s X and their own Y), correlations presented above the diagonal represent the partner associations (i.e., association between a participant’s X and their partner’s Y), and correlations in bold represent between-partner correlations (i.e., association between a participant’s X and their partner’s X).

*p < .05. *p < .01. *p < .001.

Neither internal consent feelings nor external consent communication significantly varied by gender. However, three of the five types of consent cues demonstrated effect sizes worth noting given our restricted sample size. Proportionally more women (79.3%) than men (55.6%) reported that they used explicit verbal cues during their most recent vaginal intercourse event, χ^2^(1) = 3.62, *p* = .057, φ_C_ = .25. Further, men more frequently endorsed using implicit verbal cues (66.7%) than did women (41.4%), χ^2^(1) = 3.60, *p* = .058, φ_C_ = .25; more men also reported using no response cues (29.6%) during their most recent vaginal intercourse event than did women (10.3%), χ^2^(1) = 3.29, *p* = .070, φ_C_ = .24.

Two of the internal consent feelings factors were negatively associated with relationship length. Participants who had been in their committed relationship longer reported lower levels of arousal, *r* = −.34, *p* = .011, and agreement/want, *r* = −.28, *p* = .038, during their most recent vaginal intercourse event. Relationship length was not associated with the active types of consent communication cues participants reported having used or perceived their partner had used. However, participants who had been in their committed relationship relatively longer were less likely to report having used no response cues during their most recent vaginal intercourse event, *r* = −.28, *p* = .036.

**Figure 1.**
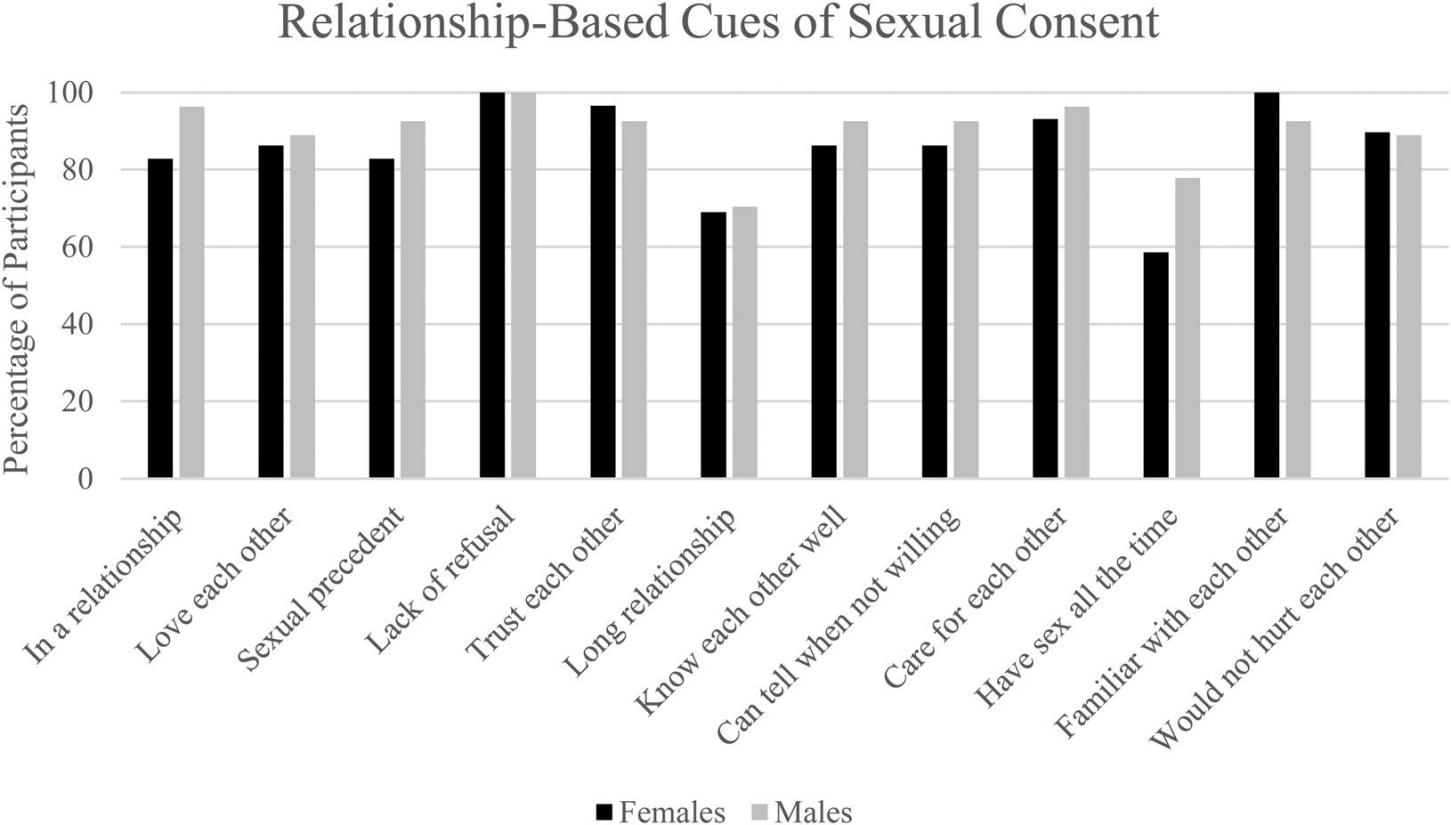
Aspects of committed relationships that participants used to perceive their partner was willing during their most recent vaginal intercourse event.

Participants overwhelmingly reported that they perceived their partner’s sexual consent during their most recent vaginal intercourse event by relying on certain context cues rather than communication cues alone (Figure 1). Of the 12 context cues we provided, 10 were endorsed by at least 87.5% of the participants. Given these high rates of endorsement and corresponding lack of variability, there were no significant differences in use of context cues based on gender. The only context cue that was associated with relationship length was “We have been together a long time,” *r* = .41, *p* = .002.

### RQ3: dyad-level sexual consent

Participants were slightly better than chance at accurately perceiving the types of sexual consent communication cues their partners self-reported using (Table 6). Almost three-quarters of participants accurately perceived whether their partner had used explicit verbal cues; accuracy was lower for each of the other cues.

Accuracy regarding sexual consent communication at the dyad-level was associated with a participant’s internal consent feelings. Participants who accurately perceived that their partner had used explicit verbal cues reported greater levels of safety/comfort (*r* = .30, *p* = .026) and agreement/want (*r* = .32, *p* = .017) during their most recent vaginal intercourse event. Further, participants whose partners accurately perceived that they had used explicit nonverbal cues reported greater levels of arousal (*r* = .31, *p* = .019) during this event. Even though there were no gender differences in self-reported or perceived cues, a participant’s use of no response cues was more accurately perceived by their partner if they were a woman than if they were a man (*r* = .40, *p* = .003). Conversely, relationship length was not associated with whether dyads accurately perceived each other’s use of any type of consent communication cue.

In a post hoc manner, we also assessed event-level orgasm as a potential correlate of sexual consent within a dyadic context. Using a logistic regression, we found that participants were 5.4 times as likely to report having experienced an orgasm if their partner used explicit verbal consent cues, *β* = 1.68, *p* = .041; they were not significantly more likely to experience multiple orgasms, *β* = .92, *p* = .347. There were no associations between experiencing orgasm and the other measured aspects of sexual consent.

## Discussion

Extending previous research on sexual consent, we collected data from dyads in committed sexual relationships and examined the potential effects of relationship length. Given the lack of previous research examining dyads and our exploratory approach, we were broad in our conceptualization of sexual consent. Specifically, we assessed all three primary aspects of sexual consent—feelings, communication, perceptions—and we considered people’s typical sexual consent experiences as well as sexual consent during their most recent vaginal intercourse event.

We found that people’s descriptions of their typical approach to sexual consent were consistent with Hickman and Muehlenhard’s (1999) conceptualization of consent communication, which has been used in several other studies (e.g., Jozkowski, Sanders, et al., 2014; Willis, Blunt-Vinti, et al., 2019). In our sample of dyads in committed relationships, participants tended to describe using consent cues that were verbal or nonverbal and explicit or implicit. Other ways that participants wrote about sexual consent in their relationship also corroborated previous research: escalation of behaviors (Muehlenhard et al., 2016), sexual behavior just happening (Willis, Canan, et al., 2020), and presence or absence of refusals (Marcantonio & Jozkowski, 2020). A novel set of themes that we considered in the present study regarded the perspective in which people described how they and their partner typically communicate their willingness to engage in sexual activity. Most participants simultaneously referred to themselves and their partners; however, some were more self-focused in their descriptions and still others partner-focused. These various perspectives may be associated with other aspects of couples’ relationships and sex lives. For example, might couple-centered conceptualizations of sexual consent communication be correlated with more adaptive outcomes (e.g., relationship satisfaction) compared with those that focus on either person?

At the event level, participants reported elevated levels of internal consent and most commonly endorsed having used explicit verbal consent cues—which aligned with the open-ended descriptions of their typical consent experiences. That participants’ most recent vaginal intercourse event was rated as highly consensual according to their responses on the ICS is consistent with previous research (e.g., Jozkowski, Sanders, et al., 2014). That we found greater endorsement of using explicit verbal cues than earlier studies on sexual consent may reflect an increased social desirability bias due to affirmative consent initiatives encouraging sexual communication that is explicit and verbal (Beres, 2014). Although Willis, Hunt, et al. (2019) found that proportionally fewer women than men reported explicit verbal cues, our data suggested that women were more likely than the men to report having used explicit verbal cues. This discrepant finding regarding gender may be due to women being more comfortable communicating consent via explicit verbal cues within a committed relationship than they would in other contexts. Indeed, Marcantonio et al. (2018) found that women with serious partners reported greater use of initiator consent communication (e.g., explicit verbal cues) than women with casual or first-time partners—other types of external sexual consent may not be as variable across relationship types.

Although previously reported associations between internal consent feelings and external consent communication have been weak to moderate, actively communicating consent verbally or nonverbally tends to be positively correlated with internal consent feelings (Jozkowski et al., 2014; Walsh et al., 2019; Willis et al., 2021). However, we did not find any significant associations between event-level internal and external sexual consent—perhaps because people in established relationships rely more on context than communication to determine whether their sexual experiences are consensual (Willis & Jozkowski, 2019). Yet, our findings suggested that accuracy regarding sexual consent communication at the dyad-level is relevant for a person’s consent feelings. Specifically, people felt elevated levels of internal consent if they (1) more accurately perceived the consent communication of their partner or (2) had a partner who more accurately perceived their consent communication. That a couple’s ability to accurately perceive each other’s sexual consent communication is associated with their internal consent emphasizes the importance of conceptualizing sexual consent as an ongoing process that should be reciprocal and mutual.

In this sample of dyads, having been in their current committed relationship for a longer amount of time was associated with relatively lower levels of internal consent feelings for participants. Because one of the feelings negatively associated with relationship length was agreement/want, we attempted to contextualize this finding within previous research on sexual compliance (i.e., consenting to unwanted sexual activity), which can occur in committed relationships when the acquiescent partner wants to please the other to show them love or to stop their nagging for sex (Willis, Fu, et al., 2020). While sexual compliance occurs to some extent in committed relationships (Katz & Schneider, 2015; Peterson & Muehlenhard, 2007), couples who have been together for relatively longer periods of time do not seem to be any more willing to engage in an unwanted consensual sexual encounter than couples who have not being together as long (Katz & Tirone, 2010; Willis & Nelson-Gray, 2020). Therefore, our finding that greater relationship length is associated with diminished internal consent may instead be due to partners relying more and more over time on context cues—rather than communication cues—to perceive, or even assume, each other’s consent (Muehlenhard et al., 2016; Righi et al., 2019; Willis & Jozkowski, 2019). Indeed, context cues were highly endorsed as indicators of sexual consent in our sample of committed dyads; however, most of these lacked adequate variability to capture any associations with relationship length. Other relational constructs (e.g., sexual satisfaction, relationship quality) may be more indicative of how partners experience and communicate their willingness to engage in sexual activity than relationship length and should therefore be included in investigations of sexual consent as a dyadic process.

Further contributing to an already mixed body of work regarding how gender is associated with internal sexual consent (Jozkowski, Peterson, et al., 2014; Walsh et al., 2019), we did not find gender differences for any of the sexual consent feelings. But consistent with previous research on external consent (Jozkowski et al., 2017; Walsh et al., 2019), women in our sample were more likely to indicate that they typically communicate their willingness by not responding or not refusing, which may be a consequence of diminished sexual agency (Tolman et al., 2015). Interestingly, at the event level men were the ones to more commonly report using no response cues. That only 2.9% of men described no response cues in their open-ended responses but 29.6% selected the corresponding closed-ended response might suggest that no response cues are not salient to men as indicators of their own consent—which could be explained by the traditionally gendered script that men view themselves as the sexual agents or pursuers of sexual activity (Curtis & Burnett, 2017; Wiederman, 2005).

## Therapeutic implications

Couples seeking therapy for help with difficulties regarding the dynamics of their sexual relationships may benefit from consent education. Basic definitional knowledge based on the empirical literature should include the three primary aspects of sexual consent: feelings, communication, and perceptions (Muehlenhard et al., 2016). For cases that present with sexual compliance or are otherwise characterized by dysfunctional sexual power differences, therapists could emphasize that sexual consent is contextual and that each partner’s willingness to engage in a particular sexual behavior at a particular point in time matters even within the context of a committed relationship. Acknowledging that consent should be prioritized in any sexual relationship, therapists should also validate people’s internal consent within a committed relationship and encourage clients not to disregard their own or their partner’s feelings of comfort, arousal, readiness, want, and physical response. Strategies for how couples might manage encounters with discrepant levels of willingness to engage in sexual activity should be considered and developed as needed.

Building on that foundation, therapists might discuss with couples how expectations regarding sexual activity and consent shift at the start of a committed relationship and can change over the course of the relationship; specifically, people tend to rely less on actively communicating consent and more on assuming it over time (Willis & Jozkowski, 2019). The findings of the present study add to these basic tenets of sexual consent by providing a first look at the importance of sexual consent perceptions in couple’s own relationships. For relationships in which sexual communication is a problem, therapists might work with couples to learn each other’s styles of communication; being able to more accurately perceive how a partner communicates their willingness to engage in sexual behavior seems to be associated with elevated levels of safety/comfort, arousal, and agreement/want—each of which reflect internal consent.

Further, researchers have posited that sexual consent is associated with relational constructs like intimacy, relationship satisfaction, and sexual pleasure (Humphreys, 2007; Marcantonio et al., 2020). For example, Satinsky and Jozkowski (2015) found that feeling entitled to sexual pleasure from a partner and being able to communicate sexual desires were both positively associated with verbally communicating consent to oral sex. Building on this work, our data suggested that participants were more likely to experience orgasm if their partner used explicit verbal consent communication cues, which provides preliminary support to claims that “consent is sexy.”

## Limitations and future directions

Investigating the dyadic nuances of sexual consent within committed relationships as we did in the present study addressed a need in the academic literature. Yet, several avenues for future research remain in light of these findings and the study’s limitations.

First, our findings may not be generalizable to populations not represented by our sample, which primarily comprised couples who identified as young, heterosexual, and White. Because most of the empirical literature on sexual consent has relied on samples with similarly homogeneous sociodemographic characteristics (Willis, Blunt-Vinti, et al., 2019), future work should assess sexual consent in dyadic samples that are more diverse regarding age, sexual orientation, and race/ethnicity. Other individual differences that would be helpful to consider include personality and ability. Further, the present work represents the experiences of people in committed sexual relationships that were monogamous; thus, the findings may not generalize to casual sexual encounters or even other types of committed sexual relationships (e.g., consensual nonmonogamy). Investigations of diverse sexual behaviors are also needed; our findings were restricted to vaginal intercourse.

Further, our analyses were underpowered; however, despite the restricted sample size, the preliminary data presented in this study were appropriate given the novelty of our exploratory research questions and will be informative for future dyadic research on sexual consent. Negatively affecting our analytic sample was that 25% of our couples did not consistently remember their most recent sexual event. As such, if researchers are interested in assessing event-level sexual consent from the perspective of two or more people, we recommend that they verify each partner is referring to the same sexual encounter before administering the sexual consent items.

Indeed, the logistical details of collecting sexual consent data from dyads require careful planning. Other salient considerations include potential privacy and safety concerns as well as an elevated propensity for responses to be affected by social desirability biases. To protect participants’ privacy in the present study, we positioned members of a dyad out of sight from each other and added blinders to the computer monitors. Even still, participants may have felt pressured to report that their most recent vaginal intercourse event with their partner aligned with various aspects of sexual consent due to the fact that participants were in the same room as their partner and knew that they were also completing the survey.

Another limitation was that the cross-sectional nature of our study prevented any claims regarding temporal effects potentially underlying the associations between sexual consent and relationship length. However, prospective longitudinal studies could provide meaningful insight regarding how sexual consent might change over the course of a committed relationship. Such study designs may be able to elucidate whether internal consent feelings actually decrease over time as our data seem to suggest. Researchers should also consider assessing sexual consent feelings, communication, and perceptions using study designs that are able to capture data more proximal to sexual activity (e.g., experience sampling methodology) to reduce memory biases inherent to sexual behavior data (Willis & Jozkowski, 2018). Collecting daily data on sexual consent from couples would also allow researchers to assess the extent that within-dyad variability exists across experiences and more precisely verify that people within a dyad are reporting on the same sexual event.

Finally, although sexual consent is often discussed in the context of sexual violence, we do not recommend extrapolating any conclusions about sexual violence based on the present data. Instead, we focused on implications regarding sexual consent within the context of consensual sexual encounters. Our declination to speculate on the implications for sexual violence should not be interpreted to mean that nonconsensual sexual activity does not occur within the context of committed sexual relationships—or is even less likely to occur. In fact, evidence consistently demonstrates that people are at even greater risk of experiencing sexual violence from intimate partners than they are from strangers or acquaintances (Peterson et al., 2021; Testa et al., 2007). Still, the present study was not designed to assess nonconsensual sexual activity. Thus, our findings are ill-equipped to provide insight on the dyadic nature of sexual willingness (or lack thereof) when one partner is being sexually assaultive.

## Conclusion

Sexual consent is a multidimensional construct that requires the participation of at least two people. By collecting data from dyads in committed sexual relationships, we provided preliminary insights regarding sexual consent feelings, communication, and perceptions that, to our knowledge, did not exist in the extant body of literature on sexual consent. Of note, we were able to measure accuracy of consent perceptions and how that is associated with consent feelings. These exploratory data suggest that further dyadic research on sexual consent is warranted. Future studies should include larger and more diverse samples and employ more sophisticated research designs to better understand dyadic-level nuances of sexual consent.

## Footnotes

1 In the present study, we conceptualized “accuracy” as whether people correctly perceived the same self-reported types of consent communication cues endorsed by their partner.

2 We did not provide participants with definitions of oral sex or vaginal intercourse, so it’s possible that participants referenced different types of behaviors (e.g., the latter may have been interpreted to include external vulvar contact, penetration with fingers, or vaginal-penile sex). Because sexual consent can vary by type of sexual behavior (Humphreys, 2007; Willis et al., 2021), researchers should more clearly define behaviors when collecting data on sexual consent.
